# AlphaFold predicts novel human proteins with knots

**DOI:** 10.1002/pro.4631

**Published:** 2023-05-01

**Authors:** Agata P. Perlinska, Wanda H. Niemyska, Bartosz A. Gren, Marek Bukowicki, Szymon Nowakowski, Pawel Rubach, Joanna I. Sulkowska

**Affiliations:** ^1^ Centre of New Technologies University of Warsaw Warsaw Poland; ^2^ Faculty of Mathematics, Informatics and Mechanics University of Warsaw Warsaw Poland; ^3^ Faculty of Physics University of Warsaw Warsaw Poland; ^4^ Warsaw School of Economics Warsaw Poland

**Keywords:** biological function, evolution, folding, new knotted folds

## Abstract

The fact that proteins can have their chain formed in a knot is known for almost 30 years. However, as they are not common, only a fraction of such proteins is available in the Protein Data Bank. It was not possible to assess their importance and versatility up until now because we did not have access to the whole proteome of an organism, let alone a human one. The arrival of efficient machine learning methods for protein structure prediction, such as AlphaFold and RoseTTaFold, changed that. We analyzed all proteins from the human proteome (over 20,000) determined with AlphaFold in search for knots and found them in less than 2% of the structures. Using a variety of methods, including homolog search, clustering, quality assessment, and visual inspection, we determined the nature of each of the knotted structures and classified it as either knotted, potentially knotted, or an artifact, and deposited all of them in a database available at: https://knotprot.cent.uw.edu.pl/alphafold. Overall, we found 51 credible knotted proteins (0.2% of human proteome). The set of potentially knotted structures includes a new complex type of a knot not reported in proteins yet. That knot type, denoted 6_3_ in mathematical notation, would necessitate a more complex folding path than any knotted protein characterized to date.

## 1 INTRODUCTION

The AlphaFold method (Jumper et al., 2021) has already led to high‐quality predictions for thousands of protein structures from different genomes. AlphaFold's approach bypasses the need to understand the complex rules that determine the physical protein‐folding process. An in silico approach can lead to the prediction of new folds and structures which are hard to determine using approaches such as crystallization NMR or cryo‐EM. AlphaFold uses a per‐residue confidence score (pLDDT) to estimate the quality of prediction. However, is the pLDDT score sufficient for quality assessment, especially in the case of long multi‐domain proteins? Since it is known that the probability of a linear chain being knotted increases with its length, can folding rules be simply bypassed? Are these longer protein structures more complicated than we can currently model accurately?

The fact that proteins can have their chain formed into a knot has been known for almost 30 years (Mansfield, n.d.; Mansfield, 1997; Takusagawa & Kamitori, 1996). Topology, and more specifically knot theory, provides tools to recognize different types of knots (e.g., Alexander and HOMFLY‐PT polynomials [Alexander, 1928; Freyd et al., 1985; Przytycki & Traczyk, 2016]). Although knot theory defines a knot as a “closed curve”, we can still study knots in proteins by connecting their N and C ends to use knot theory methods (Millett et al., 2013). So far, five types of knots have been found in experimentally solved protein structures (besides unknot these are 3_1_, 4_1_, 5_2_, and 6_1_ prime knots [Jamroz et al., 2015; Dabrowski‐Tumanski et al., 2019) and 3_1_#3_1_ complex knot (Bruno da Silva et al., 2023]). All of these prime knots are twist knots which means that they can be easily tied by twisting a loop of an open chain, pulling one end of the chain through the loop, and connecting the ends. Does AlphaFold predict protein structures with other types of knots?

Herein, we show the advantages of considering the topology of a model generated by a protein structure prediction method. In most cases, the presence of knots in proteins is conserved within a family, thus the topology of the predicted model should be the same as its homologs. Otherwise, there is a strong probability that the model was incorrectly predicted.

Furthermore, the topological analysis of proteins, especially human ones, can lead to the understanding of how evolution coded such high levels of protein organization (Sulkowska, 2020). Recent studies have shown that some proteins form open knots in their native folded structure (Mallam & Jackson, 2005; Mansfield, 1994; Sulkowska, 2020). These protein knots resemble well‐known rope knots (Sułkowska et al., 2012). In general, the percentage of known knotted proteins is much lower than would be expected in random polymers with a similar length, compactness, and flexibility (Virnau et al., 2006). Among known protein structures only the simplest of knot types have been observed (Jamroz et al., 2015) and, furthermore, only ones which can be constructed by single threading (Taylor, 2000). Numerical simulation has shown that many proteins can self‐tie (a Beccara et al., 2013; Li et al., 2012; Noel et al., 2013; Sułkowska et al., 2009) (even a protein with the 6_1_ knot (Bölinger et al., 2010)), and knotting can be assisted by a chaperone (Soler et al., 2016) or ribosome (Baiesi et al., 2019; Chwastyk & Cieplak, 2015; Dabrowski‐Tumanski et al., 2018), especially in the case of deeply knotted proteins (Jamroz et al., 2015). An in vitro investigation additionally has shown that some proteins can self‐tie (Wang, Chen, & Hsu, 2015), although chaperones facilitate their folding (Andrews et al., 2013; Jackson et al., 2017; King et al., 2010; Mallam & Jackson, 2012; Wang, Liu, et al., 2015; Ziegler et al., 2016). Interestingly, although its biological role is not clear (Jackson et al., 2017; Sulkowska, 2020), knotting has been found in proteins from all branches of the tree of life and is conserved even among the sequences with low similarity (Sułkowska et al., 2012). The presence of knots in proteins raises many fundamental questions, from which we list here just a few: (1) Is the small percentage of knotted proteins deposited in the PDB observed due to the specificity of the data? (2) Are there more complex knots which have yet to appear in the PDB? (3) What is the origin of knotted proteins? (4) Is topology strictly conserved? (5) What role do knots play in proteins?

Based on human protein structures predicted by AlphaFold, we have found answers to some of these questions. We have conducted a comprehensive review of all 23,391 structures predicted by AlphaFold for the human proteome. For each protein structure, we determined the dominant knot type (Jamroz et al., 2015) and found the location of the knot cores (i.e., minimal portions of the protein backbone that form a given knot type). As a result, we found 340 knotted structures that we then further analyzed. All these proteins are deposited in an online database to enable further in vivo and in silico investigation, available at: https://knotprot.cent.uw.edu.pl/alphafold. They are also available in AlphaKnot database alongside with knotted proteins of 20 other proteomes at https://alphaknot.cent.uw.edu.pl . (Niemyska et al., 2022 )

After careful evaluation, we concluded that over 75% of the models containing knots are artifacts. However, most of the previously known knotted human proteins were correctly predicted, which shows that AlphaFold is capable of modeling structures with complicated topology (see Table S1 for details about all proteins we classified as knotted). Compelling evidence for this is shown by a bacterial protein from *Aquifex aeolicus*, which AlphaFold models with correct knotted topology even though the single template available (TrmD protein, PDB ID: 1oy5, UniProtKB ID: O67463) is unknotted. On the other hand, it is not always the case since there are examples of errors in AlphaFold models that are due to problems in the templates from the PDB (Tata et al., 2022).

All the structures that were not classified as artifacts were put through a more detailed evaluation that included a topology analysis of homologous proteins found in the AlphaFold database. Using sequence clustering we grouped the proteins by similarity and checked if the topology is conserved both within each cluster and within the whole family of homologs. As a result, we obtained two additional pieces of information: (1) further verification of a given knot's presence in the human protein; and (2) whether the knot is robustly present (conserved) in each family. Interestingly, these results also emphasized the models that were of good quality but probably of the wrong topology. Since we obtained the models from an early version of AlphaFold Protein Structure Database we recalculated problematic structures by our locally installed AlphaFold 2 version. For example, the model of Testis anion transporter 1 protein (UniProtKB ID: Q96RN1) available in the AlphaFold Protein Structure Database v.1 has a 3_1_ knot (with high average pLDDT for the knot core being 86.1), whereas 96% of its homologs are unknotted. After recalculation with AlphaFold 2, we obtained a more credible unknotted model of this protein. Finally, for additional verification of the knotted models, we used RoseTTaFold to predict their structures and compare topologies.

Here, we established how many knotted proteins are predicted to be in a single proteome and found new families of knotted proteins. Recently, an article searching for the most complex knot types was published (Brems et al., 2022). It also analyzes AlphaFold predicted structures (including human proteins) and the topology results agree with ours. However, this extensive survey focuses on the most complex knot types and does not report any new knotted proteins with less than five crossings nor with chains longer than 600 aa nor with shallow knots (with tails shorter than 5 aa) nor with low quality (pLDDT lower than 80) (Brems et al., 2022). In our study, we find that all newly identified knotted human proteins are 3_1_ knotted.

## 2 TYPES OF PROTEIN KNOTS FOUND

Thus far four main types of knots have been observed in experimentally solved proteins—3_1_, 4_1_, 5_2_, and 6_1_ (_Jamroz et al.,_ 2015). Here, we have found three types—3_1_, 5_2_, and 6_3_ (Table 1). The 6_3_ knot has not been seen before in a protein. Note that the 4_1_ and 6_1_ knots that have not been detected in our study were previously observed mostly in bacterial and plant proteomes (16 proteins with 4_1_ and two with 6_1_).

**TABLE 1:** Number of knotted structures with different knot types found within the human proteome based on AlphaFold prediction.

Knot types	No. knotted structures	No. potentially knotted	No. artifact knots
31	49	16	189
41	0	1	12
51	0	0	10
52	2	1	17
62	0	0	3
63	0	1	0
Other	0	0	41
All	51	18	271

### 2.1 New 3_1_ knotted proteins

Overall, we found 51 credible (robustly) knotted structures. Within this group, there are proteins already known as knotted because either their structure was resolved experimentally, or they are part of an established knotted family (e.g., SPOUT clan of knotted methyltransferases). Interestingly, in some cases, we were able to find knots in proteins considered unknotted before due to their incomplete crystal structure (as in the Ion channel regulator).

The remaining proteins make a group of newly identified knotted proteins (Table 2). All of them contain a subchain forming the 3_1_ knot which is overall the most common knot type found in proteins.

**TABLE 2:** Proteins newly identified as knotted. Upper part: proteins with conserved knots found in models predicted by AlphaFold. Note that the topology matches between structures modeled by different softwares. Lower part: potentially knotted proteins with good quality models. Close homologs are represented by proteins with at least 60% sequence identity. See Methods section for details regarding structure classification.

	UniprotKB ID	Knot type AlphaFold/RoseTTaFold	Protein name	Homologs with conserved topology (%)	No. homologs	Close homologs with conserved topology (%)	No. close homologs
C O N S E R V E D K N O T T E D	O95905	3_1_/3_1_	Protein ecdysoneless homolog	100	19	100	2
	Q14CN2	3_1_/3_1_	Calcium‐activated chloride channel regulator 4	100	19	100	3
	Q5W0V3	3_1_/3_1_	FHF complex subunit HOOK interacting protein 2A	100	10	100	3
	Q86V87	3_1_/3_1_	FHF complex subunit HOOK interacting protein 2B	100	11	100	2
	Q8N612	3_1_/3_1_	FHF complex subunit HOOK interacting protein 1B	87	31	100	4
	A8K7I4	3_1_/3_1_	Calcium‐activated chloride channel regulator 1	100	19	100	5
	Q9UQC9	3_1_/3_1_	Calcium‐activated chloride channel regulator 2 exchanger 4	100	19	100	2
	Q96GM8	3_1_/3_1_	Target of EGR1 protein 1	90	10	100	4
	Q8N5C7	3_1_/3_1_	TRNA‐uridine aminocarboxypropyltransferase 1	100	14	100	3
	Q8NBA8	3_1_/3_1_	TRNA‐uridine aminocarboxypropyltransferase 2	100	9	100	3
P O T E N T I A L L Y K N O T T E D	O00534	6_3_/3_1_	von Willebrand factor A domain‐containing protein 5A	32	19	75	4
	Q9Y4D8	4_1_/0_1_	E3 ubiquitin‐protein ligase HECTD4	0	0	0	0
	Q13683	3_1_/3_1_	Integrin alpha‐7	39	90	100	2
	P23229	3_1_/3_1_	Integrin alpha‐6	38	95	100	2
	P53708	3_1_/3_1_	Integrin alpha‐8	37	95	100	4
	P06756	3_1_/3_1_	Integrin alpha‐V	39	92	100	4
	P08648	3_1_/0_1_	Integrin alpha‐5	39	94	67	3
	P26006	3_1_/0_1_	Integrin alpha‐3	38	95	100	4
	P08514	3_1_/0_1_	Integrin alpha‐IIb	39	95	100	2
	Q13075	3_1_/0_1_	Baculoviral IAP	100	8	100	6
	Q8TE82	3_1_/0_1_	SH3 domain and tetratricopeptide repeat‐containing protein 1	100	7	100	2
	Q8TF17	3_1_/0_1_	SH3 domain and tetratricopeptide repeat‐containing protein 2 subfamily U member 1	100	7	100	2
	Q9NYU2	3_1_/0_1_	UDP‐glucose: glycoprotein glucosyltransferase 1	53	15	100	2
	Q7RTX0	3_1_/0_1_	Taste receptor type 1 member 3	4	325	100	6
	Q9NQV8	3_1_/0_1_	PR domain zinc finger protein 8	100	3	100	2
	Q6UXX5	3_1_/0_1_	Inter‐α‐trypsin inhibitor H6	0	0	0	0
	Q5CZC0‐F17	3_1_/0_1_	Fibrous sheath‐interacting protein 2	0	0	0	0
	Q709C8‐F1	3_1_/0_1_	Vacuolar protein sorting‐associated protein 13C	0	0	0	0

### 2.2 Ecdysoneless protein

We found the human Ecd (ecdysoneless) protein (UniProtKB: O95905) to have been modeled by AlphaFold with a deep 3_1_ knot. (The same topology is formed in the models from RoseTTaFold). This 644 amino acid long structure has the knot positioned between 208 and 314 amino acid with average pLDDT for this region being 93/100. The knot is conserved in the homologs of the protein, including the original *Dropsophila melanogaster* Ecdysoneless protein (UniProtKB ID: Q9W032) from which the name was derived. None of the homologous proteins have structures resolved experimentally, including the whole SGT1 family (Pfam ID: PF07093) that they are part of. This makes the knot identification in this protein even more important, since SGT1 is a large family that contains over 2000 sequences. In human cells, Ecd was found to function as a regulator of the tumor suppressor p53 protein (Zhang et al., 2006). Also, the deficiency of Ecd lowers the fidelity of mRNA splicing (Erkelenz et al., 2021).

### 2.3 Ion channel regulator

**FIGURE 1:**
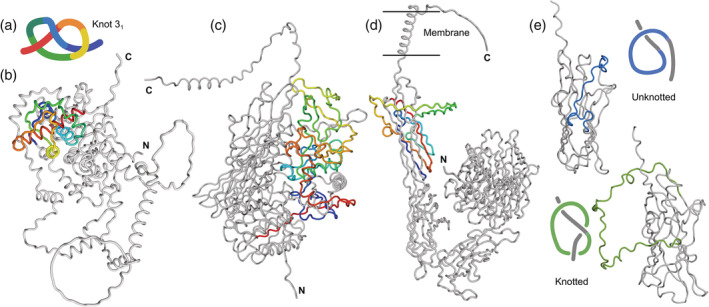
Selected proteins with a 3_1_ knot. (a) Simplified representation of a 3_1_ knot found in proteins. (b) Ecdysoneless protein (UniProtKB: O95905). (c) Calcium‐activated chloride channel regulator 1 (UniProtKB ID: A8K7I4). (d) Integrin alpha IIb (UniProtKB ID: P08514). All knotted cores are shown in a rainbow color scheme to guide the eye. (e) Upper panel: unknotted integrin alpha 1 (UniProtKB ID: P56199). Lower panel: 3_1_ knotted integrin alpha 3 (UniProtKB ID: P26006).

Calcium‐activated chloride channel regulator 1 (CLCA1; UniProtKB ID: A8K7I4) is a 914 amino acid long protein with a 3_1_ knot in its structure modeled by AlphaFold. Interestingly, this protein was resolved experimentally, although only one domain was crystallized (domain VWA, between residues 303–459). Also, this domain by itself is unknotted—the knot is present only in a full‐length protein since it is located at residues 76–311 (Figure 1b). (Note, that when all subchains are considered the internal knot [slipknot] can be detected. This motif will be called K3_1_3_1_). The knotted core is marked by AlphaFold as a high‐confidence region (average pLDDT 92.4/100). Moreover, we found that paralogs of this protein (also predicted by AlphaFold: CLCA2 and CLCA4) and other homologs all have the same knot type. Similarly, the models calculated by RoseTTaFold also have a 3_1_ knot. This shows that the prediction of a knot in this group of proteins is credible and the knot itself is a conserved feature that is likely to be advantageous for the protein.

CLCA proteins undergo self‐cleavage (CLCA1 at position 695) to form the N‐ and C‐terminal parts of the proteins. The N‐terminal portion is responsible for the function of the protein, which is activating ion channels (Yurtsever et al., 2012). Based on our analysis we now know that this part is knotted, which was not previously observed. This information will shed new light onto the studies of CLCA proteins. Moreover, this is a great example that emphasizes the importance of having full structure information available for any protein studies.

### 2.4 Knot or not?

We placed many constraints on when a model should be described as knotted (see Methods section). The structures that only met some were labeled as *potentially knotted*—usually due to either a low level of confidence in crucial parts of the knotted core or the topology not being conserved within the protein family. Those structures require further validation. In fact, this group forms a ready‐to‐use list of the most important protein targets for finding new knotted motifs, and thus can be used to significantly broaden the knowledge of entanglement in proteins (Table 2).

### 2.5 Potentially knotted—6_3_ knot in BCSC‐1

**FIGURE 2:**
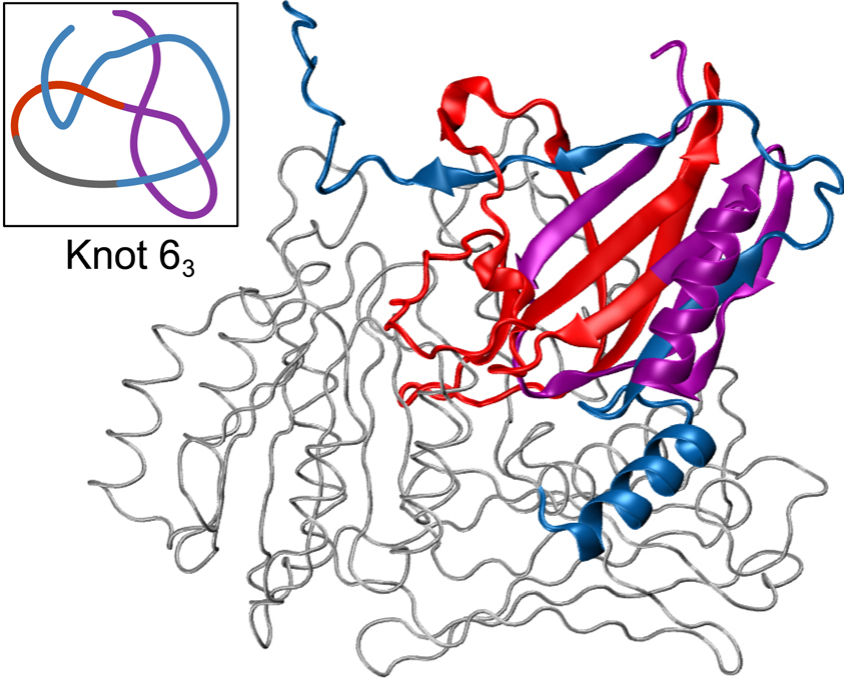
Von Willebrand factor A domain‐containing protein 5A (UniProtKB ID: O00534) with a potential new type of protein knot*—*6_3_ shown with essential strands colored in red, blue, and purple. Upper left panel shows a simplified knotted core of the protein.

We found a new complex type of knot in the von Willebrand factor A domain‐containing protein 5A (BCSC‐1, breast cancer suppressor candidate 1, Figure 2). The 6_3_ knot is located in a high confidence region (average per‐residue confidence score [pLDDT] is 88.6) between amino acids 45 and 625. It covers most of the protein, which is 786 residues long. However, because it has quite long tails (which consist of 45 and 161 amino acids, respectively), it is considered to be located rather deep within the model. This suggests that the knot will be stable in this structure and will not be untied spontaneously by thermal fluctuations of the protein.

Even though the model presents itself as accurate based on pLDDT, its 6_3_ knot type is not conserved in other species. AlphaFold predicts both 6_3_ and 3_1_ knots within the BCSC‐1 group of homologs (Table 2). Moreover, the model generated with RoseTTaFold also has a 3_1_ knot. Finally, the models generated by ESMfold also have both 6_3_ and 3_1_ knots. Therefore, further experimental examination is needed to verify the existence of this complex knot, especially since this is not a twist knot (i.e., is not the result of twisting of a loop followed by a single threading [Taylor, 2000]). To tie such a knot, the protein chain must cross the energy barrier at least twice during folding as it is pulled through twisted loops. None of the experimentally resolved structures in PDB possess this type of knot. Right now, they are only found in predicted structures—here we report a 6_3_ and recently 5_1_ and 7_1_ were found (Brems et al., 2022).

The BCSC‐1 protein is interesting not only due to its complex topology, but also because of its crucial function—there are studies showing that it is involved in cancer development and can act as its suppressor (Di et al., 2018).

### 2.6 Potentially knotted—integrins alpha

Often experimentally resolved structures have missing fragments that are not structurally organized. Such regions can be crucial for forming a knot since the way they will be modeled into the structure can either make a knot or not. The integrins alpha are a great example that shows the importance of this aspect, since within their models predicted by AlphaFold we found both knotted and unknotted structures.

We detected the 3_1_ knot in seven out of 18 human integrins alpha (Table 2). In each of them, the knot is located in the C‐terminal part of the protein (in the domain called Calf2; Figure 1C). Based on the AlphaFold predicted structures, it appears that whether an integrin will be knotted is determined by a single loop. Only integrins with longer loops are knotted (green region in Figure 1D). Unfortunately, this loop in most of the structures has a low confidence (pLDDT) score, making its location uncertain. Similarly, the topology of integrins' homologs is also mixed, making topology difficult to assess.

Therefore, we cannot verify whether the knot is present in these proteins—experimental evidence is needed. However, if only some of the integrins are indeed knotted, integrins would provide a second example of a family containing topologically distinct proteins—the first being aspartate/ornithine carbamoyltransferases (Sułkowska et al., 2008).

## 3 CONSERVED KNOTTED PROTEINS

Table 3 shows proteins for which an entangled structure of a distinct homolog is known, and thus they are expected to be knotted. Most of them belong to four families (UCH, SPOUT, sodium/calcium exchanger, carbonic anhydrase‐related). First, let us discuss transmembrane proteins, for which the knot conservation we found most interesting. Herein, all eight transmembrane proteins belong to only two families SLC24 and SLC8 (sodium/calcium exchanger). They all share a 9‐helix inverted repeat motif, which forms a transmembrane channel, its active part 6‐helix responsible for ion transport forms a 3_1_ knot. SLC24 and SLC8 represent distant homologs of only one known family of knotted membrane proteins (represented by CAX, NCX) (Jarmolinska et al., 2019). These proteins belong to a huge membrane family PF01699, represented by more than 47,000 sequences. In this context, one is tempted to speculate whether all members possess nontrivial topology. Nevertheless, the 3_1_ knot is conserved between SLC24, SLC8, and CAX, NCX proteins which share less than 30% similarity. Evolutionary conservation of a knotted topology, in this case, could imply its beneficial role, as it has been suggested for slipknotted membrane proteins (which also belong to Alpha‐helical polytopic Transmembrane classes (based on OPM classification; Zayats et al., 2021). Therein, it was suggested that the channel may be held together more tightly when its α‐helices are strapped together by a slipknot loop embracing several of the helices (Sułkowska et al., 2012).

**TABLE 3:** Proteins expected to be entangled. Here are the proteins that do not have a resolved structure but are part of a known family of knotted proteins, thus have a high probability of being correctly predicted as knotted. Close homologs are represented by proteins with at least 60% sequence identity.

UniprotKB ID	Knot type (AlphaFold)	Protein name	Homologs with conserved topology (%)	No. homologs	Close homologs with conserved topology (%)	No. close homologs
Q92560	5_2_	Ubiquitin carboxyl‐terminal hydrolase BAP1	88	8	75	4
Q6PF06	3_1_	tRNA methyltransferase 10 homolog B	100	41	100	3
Q6IN84	3_1_	rRNA methyltransferase 1, mitochondrial	96	28	100	2
Q9UJK0	3_1_	18S rRNA aminocarboxypropyltransferase	81	37	100	3
P32418	3_1_	Sodium/calcium exchanger 1	100	36	100	7
Q9UPR5	3_1_	Sodium/calcium exchanger 2	100	36	100	9
P57103	3_1_	Sodium/calcium exchanger 3	100	36	100	9
Q9UI40	3_1_	Sodium/potassium/calcium exchanger 2	87	46	100	1
Q9HC58	3_1_	Sodium/potassium/calcium exchanger 3	91	67	100	2
Q8NFF2	3_1_	Sodium/potassium/calcium exchanger 4	88	34	50	2
Q71RS6	3_1_	Sodium/potassium/calcium exchanger 5	88	42	100	3
Q6J4K2	3_1_	Mitochondrial sodium/calcium exchanger protein	91	69	100	3
O75493	3_1_	Carbonic anhydrase‐related protein 11	99	156	100	6
Q9NS85	3_1_	Carbonic anhydrase‐related protein 10	99	159	100	7
Q9Y2D0	3_1_	Carbonic anhydrase 5B, mitochondrial	98	172	100	3
P35218	3_1_	Carbonic anhydrase 5A, mitochondrial	95	181	100	3

Second, ubiquitin carboxyl‐terminal hydrolase BAP1 (UCH‐L2) with a 5_2_ knot is a distant homolog of other UCH proteins. All homologs (UCHL1, UCHL3, UCHL5, UBL1‐YEAST) with known structures possess the same type of topology (including internal 3_1_ knots), even though their sequence similarity is very low, around 23% (Sułkowska et al., 2012). BAP1 is known to play a role in cancer, functioning both as a tumor suppressor and as a metastasis suppressor.

Third, there are plenty of carbonic anhydrase‐related proteins already deposited in the PDB, they all are found to possess a 3_1_ knot when a full sequence is determined. Finally, proteins with the UniProtKB ID: Q6PF06, Q6IN84, Q9UJK0 are members of SPOUT family (Tkaczuk et al., 2007), which is known to contain proteins with a conserved 3_1_ knot embedded in the active site (Christian et al., 2016).

### 3.1 Artifacts

More than 75% of knotted human structures from AlphaFold we found to be artifacts. In such structures, the knot is often predicted in low confidence regions (pLDDT < 50), which means its position and presence is not reliable. Moreover, about 1/3 of the structures with artifact knots represent large proteins (>2700 aa) that were predicted using multiple overlapping models. In fact, more than 50% of the artifact structures, versus only 17% of the credibly knotted structures, have more than 1000 residues. This behavior is expected, since the difficulty of predicting a structure (including its topology) increases with the length of the protein chain. Thus, topology can be used as a tool to identify the quality of experimental or simulated data. Some examples of interesting artifacts are described on the website.

## 4 CONCLUSION

While knots are relatively rare in proteins, their conservation may suggest a functional utility. Many of them are enzymes with a knot located in the active site. However, as they are not common, only a fraction of such proteins is available in the PDB. It was not possible to assess their importance and versatility up until now because we did not have access to the whole proteome of an organism, let alone a human one. The arrival of efficient machine learning methods for protein structure prediction, such as AlphaFold, RoseTTaFold, and ESMFold changed that.

Herein, we analyzed all proteins from human proteome (over 20,000) determined with AlphaFold in search for knots and found them in less than 2% of the structures. Information about their topology, knot position, and quality (pLDDT score) is available in a database at: https://knotprot.cent.uw.edu.pl/alphafold. Importantly, after detailed analysis we found that the majority of the knotted proteins are artifacts and only 51 structures (0.2% of the proteome) are credibly knotted.

The set of credibly knotted proteins includes new knotted families and new types of knots (Table 2). The set of potentially knotted structures includes a new complex knot type in proteins. That knot type, denoted 6_3_ in mathematical notation, would necessitate a more complex folding path than any knotted protein characterized to date.

To sum up, we select proteins with the best chance of being entangled, and which can be further analyzed without immediate structure determination. However, it would be beneficial to verify experimentally the structures of several proteins, including those of uncertain topology (Table 2), like integrins alpha. Also, our work emphasizes the importance of validation of the predicted models, as pLDDT in some cases is not sufficient to distinguish between correct and wrong models, in particular in terms of their topology.

## 5 MATERIALS AND METHODS

The human proteome was downloaded from the AlphaFold Protein Structure Database (Jumper et al., 2021). It consisted of 23,391 structures (20,504 proteins). All the models were analyzed by computing the HOMFLY‐PT polynomial for 200 random closures. The details of the method are explained in (Jamroz et al., 2015). We determined the position of the knot core in the structure based on the so‐called matrix model (Jamroz et al., 2015). Homologs for each protein were obtained with GGSearch from EMBL‐EBI (Madeira et al., 2022) using E‐value cutoff 10^−10^ and searching within the AlphaFold Protein Structure Database. For the analysis of the conservation of topology in protein families, we clustered the proteins using CD‐hit with a 60% sequence identity cutoff (Huang et al., 2010). Proteins with very low pLDDT were remodeled by our group using AlphaFold (version 2.1.1) (Jumper et al., 2021), with the program installed locally. For the RoseTTaFold prediction, we used software available on‐line (Baek et al., 2021).

A structure with a knot is classified as credible when random closures form a nontrivial knot type more frequently than a trivial knot, the pLDDT score for the knot core is above 50, there are no clashes in the knot core region (checked with MolProbity tool [Williams et al., 2018]), the same knot type is found in more than 80% of the protein's homologs and in a model generated by RoseTTaFold, and no suspicious geometry was found after visual inspection. The structures which did not pass the visual inspection were classified as potentially knotted. The structures with obvious problems are called artifacts.

## AUTHOR CONTRIBUTIONS

**Agata Paulina Perlińska:** Formal analysis (equal); investigation (equal); methodology (equal); validation (equal); visualization (equal); writing – original draft (equal); writing – review and editing (equal). **Wanda Niemyska:** Data curation (equal); validation (equal); investigation (equal); methodology (equal);writing – review and editing (equal). **Bartosz Greń:** Data curation (equal), investigation (equal); methodology (equal); software (equal); visualization (equal). **Marek Bukowicki:** Software (equal). **Szymon Nowakowski:** Software (equal). **Paweł Rubach:** Data curation (equal); investigation (equal); methodology (equal); software (equal); writing – review and editing (equal). **Joanna Sulkowska:** Formal analysis (equal); investigation (equal); validation (equal); writing – original draft (equal); writing – review and editing (equal).

## CONFLICT OF INTEREST STATEMENT

The authors declare no conflicts of interest.

## Supporting information


**Table S1:** All knotted proteins found within human proteome.

## References

[pro4631-bib-0001] a Beccara S , Škrbić T , Covino R , Micheletti C , Faccioli P . Folding pathways of a knotted protein with a realistic atomistic force field. PLoS Comput Biol. 2013;9(3):e1003002. 23555232 10.1371/journal.pcbi.1003002 PMC3605060

[pro4631-bib-0002] Alexander JW . Topological invariants of knots and links. Trans Am Math So. 1928;30(2):275–306.

[pro4631-bib-0003] Andrews BT , Capraro DT , Sulkowska JI , Onuchic JN , Jennings PA . Hysteresis as a marker for complex, overlapping landscapes in proteins. J Phys Chem Lett. 2013;4(1):180–8. 23525263 10.1021/jz301893w PMC3601837

[pro4631-bib-0004] Baek M , DiMaio F , Anishchenko I , Dauparas J , Ovchinnikov S , Lee GR , et al. Accurate prediction of protein structures and interactions using a three‐track neural network. Science. 2021;373(6557):871–6. 34282049 10.1126/science.abj8754 PMC7612213

[pro4631-bib-0005] Baiesi M , Orlandini E , Seno F , Trovato A . Sequence and structural patterns detected in entangled proteins reveal the importance of co‐translational folding. Sci Rep. 2019;9(1):1–2. 31182755 10.1038/s41598-019-44928-3 PMC6557820

[pro4631-bib-0006] Bölinger D , Sułkowska JI , Hsu HP , Mirny LA , Kardar M , Onuchic JN , et al. A Stevedore's protein knot. PLoS Comput Biol. 2010;6(4):e1000731. 20369018 10.1371/journal.pcbi.1000731 PMC2848546

[pro4631-bib-0007] Brems MA , Runkel R , Yeates TO , Virnau P . AlphaFold predicts the most complex protein knot and composite protein knots. Protein Sci. 2022;31(8):e4380. 35900026 10.1002/pro.4380 PMC9278004

[pro4631-bib-0008] Bruno da Silva F , Lewandowska I , Kluza A , Niewieczerzal S , Augustyniak R , Sulkowska JI . First crystal structure of double knotted protein TrmD‐Tm1570*—*inside from degradation perspective. bioRxiv. 2023; 532328.

[pro4631-bib-0009] Christian T , Sakaguchi R , Perlinska AP , Lahoud G , Ito T , Taylor EA , et al. Methyl transfer by substrate signaling from a knotted protein fold. Nat Struct Mol Biol. 2016;23(10):941–8. 27571175 10.1038/nsmb.3282 PMC5429141

[pro4631-bib-0010] Chwastyk M , Cieplak M . Cotranslational folding of deeply knotted proteins. J Phys Condens Matter. 2015;27(35):354105. 26292194 10.1088/0953-8984/27/35/354105

[pro4631-bib-0011] Dabrowski‐Tumanski P , Piejko M , Niewieczerzal S , Stasiak A , Sulkowska JI . Protein knotting by active threading of nascent polypeptide chain exiting from the ribosome exit channel. J Phys Chem B. 2018;122(49):11616–25. 30198720 10.1021/acs.jpcb.8b07634

[pro4631-bib-0012] Dabrowski‐Tumanski P , Rubach P , Goundaroulis D , Dorier J , Sułkowski P , Millett KC , et al. KnotProt 2.0: a database of proteins with knots and other entangled structures. Nucleic Acids Res. 2019;47(D1):D367–75. 30508159 10.1093/nar/gky1140 PMC6323932

[pro4631-bib-0013] Di D , Chen L , Guo Y , Wang L , Wang H , Ju J . Association of BCSC‐1 and MMP‐14 with human breast cancer. Oncol Lett. 2018;15(4):5020–6. 29552138 10.3892/ol.2018.7972 PMC5840690

[pro4631-bib-0014] Erkelenz S , Stanković D , Mundorf J , Bresser T , Claudius AK , Boehm V , et al. Ecd promotes U5 snRNP maturation and Prp8 stability. Nucleic Acids Res. 2021;49(3):1688–707. 33444449 10.1093/nar/gkaa1274 PMC7897482

[pro4631-bib-0015] Freyd P , Yetter D , Hoste J , Lickorish WR , Millett K , Ocneanu A . A new polynomial invariant of knots and links. Bulletin (new series) of the American mathematical. Society. 1985;12(2):239–46.

[pro4631-bib-0016] Huang Y , Niu B , Gao Y , Fu L , Li W . CD‐HIT suite: a web server for clustering and comparing biological sequences. Bioinformatics. 2010;26(5):680–2. 20053844 10.1093/bioinformatics/btq003 PMC2828112

[pro4631-bib-0017] Jackson SE , Suma A , Micheletti C . How to fold intricately: using theory and experiments to unravel the properties of knotted proteins. Curr Opin Struct Biol. 2017;42:6–14. 27794211 10.1016/j.sbi.2016.10.002

[pro4631-bib-0018] Jamroz M , Niemyska W , Rawdon EJ , Stasiak A , Millett KC , Sułkowski P , et al. KnotProt: a database of proteins with knots and slipknots. Nucleic Acids Res. 2015;43(D1):D306–14. 25361973 10.1093/nar/gku1059 PMC4383900

[pro4631-bib-0019] Jarmolinska AI , Perlinska AP , Runkel R , Trefz B , Ginn HM , Virnau P , et al. Proteins' knotty problems. J Mol Biol. 2019;431(2):244–57. 30391297 10.1016/j.jmb.2018.10.012

[pro4631-bib-0020] Jumper J , Evans R , Pritzel A , Green T , Figurnov M , Ronneberger O , et al. Highly accurate protein structure prediction with AlphaFold. Nature. 2021;596(7873):583–9. 34265844 10.1038/s41586-021-03819-2 PMC8371605

[pro4631-bib-0021] King NP , Jacobitz AW , Sawaya MR , Goldschmidt L , Yeates TO . Structure and folding of a designed knotted protein. Proc Natl Acad Sci. 2010;107(48):20732–7. 21068371 10.1073/pnas.1007602107 PMC2996448

[pro4631-bib-0022] Li W , Terakawa T , Wang W , Takada S . Energy landscape and multiroute folding of topologically complex proteins adenylate kinase and 2ouf‐knot. Proc Natl Acad Sci. 2012;109(44):17789–94. 22753508 10.1073/pnas.1201807109 PMC3497823

[pro4631-bib-0023] Madeira F , Pearce M , Tivey AR , Basutkar P , Lee J , Edbali O , et al. Search and sequence analysis tools services from EMBL‐EBI in 2022. Nucleic Acids Res. 2022;50(W1):W276–9. 35412617 10.1093/nar/gkac240 PMC9252731

[pro4631-bib-0024] Mallam AL , Jackson SE . Folding studies on a knotted protein. J Mol Biol. 2005;346(5):1409–21. 15713490 10.1016/j.jmb.2004.12.055

[pro4631-bib-0025] Mallam AL , Jackson SE . Knot formation in newly translated proteins is spontaneous and accelerated by chaperonins. Nat Chem Biol. 2012;8(2):147–53. 10.1038/nchembio.742 22179065

[pro4631-bib-0026] Mansfield ML . Are there knots in proteins? Nat Struct Biol. 1994;1(4):213–4. 7656045 10.1038/nsb0494-213

[pro4631-bib-0027] Mansfield ML . Fit to be tied. Nat Struct Biol. 1997;4(3):166–7. 9164450 10.1038/nsb0397-166

[pro4631-bib-0028] Millett KC , Rawdon EJ , Stasiak A , Sułkowska JI . Identifying knots in proteins. Biochem Soc Trans. 2013;41(2):533–7. 23514149 10.1042/BST20120339

[pro4631-bib-0029] Niemyska W , Rubach P , Gren BA , Nguyen ML , Garstka W , Bruno da Silva F , et al. AlphaKnot: server to analyze entanglement in structures predicted by AlphaFold methods. Nucleic Acids Res. 2022;50(W1):W44–50. 35609987 10.1093/nar/gkac388 PMC9252816

[pro4631-bib-0030] Noel JK , Onuchic JN , Sulkowska JI . Knotting a protein in explicit solvent. J Phys Chem Lett. 2013;4(21):3570–3.

[pro4631-bib-0031] Przytycki JH , Traczyk P . Invariants of links of Conway type. arXiv preprint *arXiv*:1610.06679 2016.

[pro4631-bib-0032] Soler MA , Rey A , Faísca PF . Steric confinement and enhanced local flexibility assist knotting in simple models of protein folding. Phys Chem Phys. 2016;18(38):26391–403. 10.1039/c6cp05086g 27722468

[pro4631-bib-0033] Sulkowska JI . On folding of entangled proteins: knots, lassos, links and θ‐curves. Curr Opin Struct Biol. 2020;60:131–41. 32062143 10.1016/j.sbi.2020.01.007

[pro4631-bib-0034] Sułkowska JI , Rawdon EJ , Millett KC , Onuchic JN , Stasiak A . Conservation of complex knotting and slipknotting patterns in proteins. Proc Natl Acad Sci. 2012;109(26):E1715–23. 22685208 10.1073/pnas.1205918109 PMC3387036

[pro4631-bib-0035] Sułkowska JI , Sułkowski P , Onuchic J . Dodging the crisis of folding proteins with knots. Proc Natl Acad Sci. 2009;106(9):3119–24. 19211785 10.1073/pnas.0811147106 PMC2651233

[pro4631-bib-0036] Sułkowska JI , Sułkowski P , Szymczak P , Cieplak M . Stabilizing effect of knots on proteins. Proc Natl Acad Sci. 2008;105(50):19714–9. 19064918 10.1073/pnas.0805468105 PMC2604914

[pro4631-bib-0037] Takusagawa F , Kamitori S . A real knot in protein. J Am Chem Soc. 1996;118(37):8945–6.

[pro4631-bib-0038] Tata RB , Alsulami AF , Sheik Amamuddy O , Blundell TL , Tastan BÖ . Slipknot or crystallographic error: a computational analysis of the plasmodium falciparum DHFR structural folds. Int J Mol Sci. 2022;23(3):1514. 35163439 10.3390/ijms23031514 PMC8835989

[pro4631-bib-0039] Taylor WR . A deeply knotted protein structure and how it might fold. Nature. 2000;406(6798):916–9. 10972297 10.1038/35022623

[pro4631-bib-0040] Tkaczuk KL , Dunin‐Horkawicz S , Purta E , Bujnicki JM . Structural and evolutionary bioinformatics of the SPOUT superfamily of methyltransferases. BMC Bioinform. 2007;8(1):1–31. 10.1186/1471-2105-8-73 PMC1829167 17338813

[pro4631-bib-0041] Virnau P , Mirny LA , Kardar M . Intricate knots in proteins: function and evolution. PLoS Comput Biol. 2006;2(9):e122. 16978047 10.1371/journal.pcbi.0020122 PMC1570178

[pro4631-bib-0042] Wang I , Chen SY , Hsu ST . Unraveling the folding mechanism of the smallest knotted protein, MJ0366. J Phys Chem B. 2015;119(12):4359–70. 25741995 10.1021/jp511029s

[pro4631-bib-0043] Wang LW , Liu YN , Lyu PC , Jackson SE , Hsu ST . Comparative analysis of the folding dynamics and kinetics of an engineered knotted protein and its variants derived from HP0242 of helicobacter pylori. J Phys Condens Matter. 2015;27(35):354106. 26290953 10.1088/0953-8984/27/35/354106

[pro4631-bib-0044] Williams CJ , Headd JJ , Moriarty NW , Prisant MG , Videau LL , Deis LN , et al. MolProbity: more and better reference data for improved all‐atom structure validation. Protein Sci. 2018;27(1):293–315. 29067766 10.1002/pro.3330 PMC5734394

[pro4631-bib-0045] Yurtsever Z , Sala‐Rabanal M , Randolph DT , Scheaffer SM , Roswit WT , Alevy YG , et al. Self‐cleavage of human CLCA1 protein by a novel internal metalloprotease domain controls calcium‐activated Chloride Channel activation*♦. J Biol Chem. 2012;287(50):42138–49. 23112050 10.1074/jbc.M112.410282 PMC3516759

[pro4631-bib-0046] Zayats V , Perlinska AP , Jarmolinska AI , Jastrzebski B , Dunin‐Horkawicz S , Sulkowska JI . Slipknotted and unknotted monovalent cation‐proton antiporters evolved from a common ancestor. PLoS Comput Biol. 2021;17(10):e1009502. 34648493 10.1371/journal.pcbi.1009502 PMC8562792

[pro4631-bib-0047] Zhang Y , Gurumurthy CB , Kim J , Bhat I , Gao Q , Dimri G , et al. The human orthologue of drosophila ecdysoneless protein interacts with p53 and regulates its function. Cancer Res. 2006;66(14):7167–75. 16849563 10.1158/0008-5472.CAN-06-0722

[pro4631-bib-0048] Ziegler F , Lim NC , Mandal SS , Pelz B , Ng WP , Schlierf M , et al. Knotting and unknotting of a protein in single molecule experiments. Proc Natl Acad Sci. 2016;113(27):7533–8. 27339135 10.1073/pnas.1600614113 PMC4941514

