# Supplementary material for: AlphaFold predicts novel human proteins with knots
Source: Protein Sci. 2023 May 1;32(5):e4631. doi: 10.1002/pro.4631 (PMC10108431; doi:10.1002/pro.4631)
Supplement: Supplementary file 1 — Table S1: All knotted proteins found within human proteome. [file PRO-32-e4631-s001.pdf]

Table S1: All knotted proteins found within human proteome.

Conservation	Known/ new	UniProtKB ID	Protein Name	Knot type (AlphaFold/ RoseTTaFold)	Knot core	Location in the cell	Pfam ID (in knot core)	PDB ID	Notes
Conserved	New	Q8N5C7	tRNA-uridine aminocarboxypropyltransferase 1	3_1/3_1	136-231	nucleus	PF03942	-	
Conserved	New	Q8NBA8	tRNA-uridine aminocarboxypropyltransferase 2	3_1/3_1	148-203	cytoplasm/ nucleus	PF03942	-	
Conserved	New	Q96GM8	Target of EGR1 protein 1	3_1/3_1	29-455	nucleolus	PF04857 - PF04857 - PF00642	2fc6	2fc6 is unknotted due to incomplete structure
Conserved	New	O95905	Protein ecdysoneless homolog	3_1/3_1	208-314	cytoplasm/ nucleus	PF07093	-	
Conserved	New	Q9UQC9	Calcium-activated chloride channel regulator 2	3_1/3_1	85-313	cell membrane	PF08434	-	
Conserved	New	Q14CN2	Calcium-activated chloride channel regulator 4	3_1/3_1	77-312	cell membrane	PF08434 - PF00092	-	
Conserved	New	A8K7I4	Calcium-activated chloride channel regulator 1	3_1/3_1	76-311	cell membrane	PF08434 - PF13519	6pyo	6pyo is unknotted due to incomplete structure
Conserved	New	Q5W0V3	FHF complex subunit HOOK interacting protein 2A	3_1/3_1	467-759	-	PF10257- PF19314- PF19311	-	
Conserved	New	Q86V87	FHF complex subunit HOOK interacting protein 2B	3_1/3_1	469-731	-	PF10257- PF19314- PF19311	-	
Conserved	New	Q8N612	FHF complex subunit HOOK interacting protein 1B	3_1/3_1	421-961	cytoplasm	PF10257- PF19314- PF19311	-	
Potential	New	Q8TE82	SH3 domain and tetratricopeptide repeat-containing protein 1	3_1/0_1	368-852	-	-	-	
Potential	New	Q8TF17	SH3 domain and tetratricopeptide repeat-containing protein 2	3_1/0_1	338-831	endosome	-	-	
Potential	New	Q13075	Baculoviral IAP repeat-containing protein 1	3_1/0_1	905-1401	cytoplasm	-	2vm5	2vm5 is unknotted due to incomplete structure
Potential	New	Q9NQV8	PR domain zinc finger protein 8	3_1/0_1	20-96	nucleus	-	-	
Potential	New	Q7RTX0	Taste receptor type 1 member 3	3_1/0_1	23-398	cell membrane	PF01094	-	
Potential	New	Q13683	Integrin alpha-7	3_1/3_1	901-1025	membrane	PF08441	-	
Potential	New	P23229	Integrin alpha-6	3_1/3_1	887-996	cell membrane	PF08441	7ceb	7ceb is unknotted due to incomplete structure
Potential	New	P53708	Integrin alpha-8	3_1/3_1	838-955	cell membrane	PF08441	-	
Potential	New	P26006	Integrin alpha-3	3_1/0_1	811-934	cell membrane	PF08441	-	
Potential	New	P06756	Integrin alpha-V	3_1/0_1	822-940	cell membrane	PF08441	1jv2	incomplete structure
Potential	New	P08514	Integrin alpha-IIb	3_1/0_1	823-946	membrane	PF08441	6v4p	incomplete structure
Potential	New	P08648	Integrin alpha-5	3_1/0_1	842-942	membrane	PF08441	7nwl	incomplete structure
Potential	New	Q6UXX5	Inter-alpha-trypsin inhibitor heavy chain H6	3_1/0_1	79-618	extracellular	PF08487 - PF00092	-	
Potential	New	O00534	von Willebrand factor A domain-containing protein 5A	6_3/3_1	45-625	nucleus	PF08487 - PF13768	-	

Conservation	Known/ new	UniProtKB ID	Protein Name	Knot type (AlphaFold/ RoseTTaFold)	Knot core	Location in the cell	Pfam ID (in knot core)	PDB ID	Notes
Potential	New	Q9NYU2	UDP-glucose:glycoprotein glucosyltransferase 1	3_1/0_1	35-1070	endoplasmic reticulum	PF18400-PF18401-PF18402-PF18403	-	
Potential	New	Q9Y4D8	Probable E3 ubiquitin-protein ligase HECTD4	4_1	629-1375 (F7)	membrane	-	-	multimodel AF prediction
Potential	New	Q709C8	Vacuolar protein sorting-associated protein 13C	3_1	101-544 (F1)	mitochondrion membrane	PF12624-PF16908	-	multimodel AF prediction
Potential	New	Q5CZC0	Fibrous sheath-interacting protein 2	3_1	1098-1234 (F17)	sperm	-	-	multimodel AF prediction
Known	Known structure	P07451	Carbonic anhydrase 3	3_1	25-259	cytoplasm	PF00194	1z93	Carbonic anhydrase family
Known	Known structure	P43166	Carbonic anhydrase 7	3_1	21-262	cytoplasm	PF00194	3ml5	Carbonic anhydrase family
Known	Known structure	O43570	Carbonic anhydrase 12	3_1	53-290	cell membrane	PF00194	4ht2	Carbonic anhydrase family
Known	Known structure	P00918	Carbonic anhydrase 2	3_1	21-259	cytoplasm/cell membrane	PF00194	4kap	Carbonic anhydrase family
Known	Known structure	Q16790	Carbonic anhydrase 9	3_1	157-391	cell membrane/ nucleus	PF00194	5dvx	Carbonic anhydrase family
Known	Known structure	Q8N1Q1	Carbonic anhydrase 13	3_1	42-281	cytoplasm	PF00194	3czv	Carbonic anhydrase family
Known	Known structure	Q9ULX7	Carbonic anhydrase 14	3_1	21-299	membrane	PF00194	4lu3	Carbonic anhydrase family
Known	Known structure	P22748	Carbonic anhydrase 4	3_1	48-285	cell membrane	PF00194	1znc	Carbonic anhydrase family
Known	Known structure	P35219	Carbonic anhydrase-related protein	3_1	35-288	cytoplasm	PF00194	2w2j	Carbonic anhydrase family
Known	Known structure	P23280	Carbonic anhydrase 6	3_1	44-282	extracellular	PF00194	3fe4	Carbonic anhydrase family
Known	Known structure	P31153	S-adenosylmethionine synthase isoform type-2	3_1	21-299	cytoplasm	PF00438 - PF02772	2p02	S-adenosylmethionine synthetase family
Known	Known structure	Q00266	S-adenosylmethionine synthase isoform type-1	3_1	7-285	cytoplasm	PF00438 - PF02772	2obv	S-adenosylmethionine synthetase family
Known	Known structure	Q9HC36	rRNA methyltransferase 3, mitochondrial	3_1	282-379	mitochondrion	PF00588	7oi6	SPOUT superfamily
Known	Known structure	Q13395	Probable methyltransferase TARBP1	3_1	1538-1586	nucleus	PF00588	2ha8	SPOUT superfamily
Known	Known structure	Q9Y5K5	Ubiquitin carboxyl-terminal hydrolase isozyme L5	5_2	6-230	cytoplasm/ nucleus	PF01088	3a7s	UCH family
Known	Known structure	Q8TBZ6	tRNA methyltransferase 10 homolog A	3_1	181-232	nucleus	PF01746	4fmw	SPOUT superfamily
Known	Known structure	Q7L0Y3	tRNA methyltransferase 10 homolog C	3_1	285-336	mitochondrion	PF01746	5nfj	SPOUT superfamily
Known	Known structure	Q5T280	Putative methyltransferase C9orf114	3_1	282-341	cytoskeleton	PF02598	4rg1	SPOUT superfamily
Known	Known structure	Q12791	Calcium-activated potassium channel subunit alpha-1	3_1	540-1106	cell membrane	PF03493	3mt5	
Known	Known structure	Q92979	Ribosomal RNA small subunit methyltransferase NEP1	3_1	171-219	nucleolus	PF03587	5fai	SPOUT superfamily
Known	Known structure	Q7RTV0	PHD finger-like domain-containing protein 5A	3_1	16-71	nucleus	PF03660	5syb	

Conservation	Known/ new	UniProtKB ID	Protein Name	Knot type (AlphaFold/ RoseTTaFold)	Knot core	Location in the cell	Pfam ID (in knot core)	PDB ID	Notes
Known	Known structure	Q9NP92	39S ribosomal protein S30, mitochondrial	3_1	224-298	mitochondrion	PF07147	3j7y	
Known	Known structure	Q9BZE1	39S ribosomal protein L37, mitochondrial	3_1	218-297	mitochondrion	PF07147	3j7y	
Known	Known structure	O95831	Apoptosis-inducing factor 1, mitochondrial	3_1	433-611	cytoplasm/mitochondrion/nucleus	PF14721	4bv6	
Known	Known structure	A8MYU2	Potassium channel subfamily U member 1	3_1/0_1	460-966	cell membrane	PF03493	4hpf	
Conserved	Known family	O75493	Carbonic anhydrase-related protein 11	3_1	66-304	extracellular	PF00194	-	Carbonic anhydrase family
Conserved	Known family	Q9NS85	Carbonic anhydrase-related protein 10	3_1	62-301	-	PF00194	-	Carbonic anhydrase family
Conserved	Known family	Q9Y2D0	Carbonic anhydrase 5B, mitochondrial	3_1	61-297	mitochondrion	PF00194	-	Carbonic anhydrase family
Conserved	Known family	P35218	Carbonic anhydrase 5A, mitochondrial	3_1	59-295	mitochondrion	PF00194	-	Carbonic anhydrase family
Conserved	Known family	Q6IN84	rRNA methyltransferase 1, mitochondrial	3_1	220-277	mitochondrion	PF00588	-	SPOUT superfamily
Conserved	Known family	Q92560	Ubiquitin carboxyl-terminal hydrolase BAP1	5_2	4-238	cytoplasm/nucleus	PF01088	-	UCH family
Conserved	Known family	Q6J4K2	Mitochondrial sodium/calcium exchanger protein	3_1	114-508	mitochondrion	PF01699 - PF01699	-	Sodium/calcium exchanger family
Conserved	Known family	Q9HC58	Sodium/potassium/calcium exchanger 3	3_1	107-548	membrane	PF01699 - PF01699	-	Sodium/calcium exchanger family
Conserved	Known family	Q71RS6	Sodium/potassium/calcium exchanger 5	3_1	64-399	Golgi membrane	PF01699 - PF01699	-	Sodium/calcium exchanger family
Conserved	Known family	Q9UI40	Sodium/potassium/calcium exchanger 2	3_1	130-565	membrane	PF01699 - PF01699	-	Sodium/calcium exchanger family
Conserved	Known family	Q8NFF2	Sodium/potassium/calcium exchanger 4	3_1/3_1	99-518	cytoplasm/cell membrane	PF01699 - PF01699	-	Sodium/calcium exchanger family
Conserved	Known family	P32418	Sodium/calcium exchanger 1	3_1	84-884	cell membrane	PF01699 - PF16494 - PF03160 - PF03160 - PF01699	-	Sodium/calcium exchanger family
Conserved	Known family	P57103	Sodium/calcium exchanger 3	3_1	87-841	cell membrane	PF01699 - PF16494 - PF03160 - PF03160 - PF01699	-	Sodium/calcium exchanger family
Conserved	Known family	Q9UPR5	Sodium/calcium exchanger 2	3_1	82-836	cell membrane	PF01699 - PF16494 - PF03160 - PF03160 - PF01699	-	Sodium/calcium exchanger family
Conserved	Known family	Q6PF06	tRNA methyltransferase 10 homolog B	3_1	208-258	cytoplasm/mitochondrion/nucleus	PF01746	-	SPOUT superfamily
Conserved	Known family	Q9UJK0	18S rRNA aminocarboxypropyltransferase	3_1	94-145	cytoplasm	PF04034	-	Ribosome biogenesis family
